# Supplementary material for: On the Properties of Styrene–Maleic Acid Copolymer–Lipid Nanoparticles: A Solution NMR Perspective
Source: Polymers (Basel). 2024 Oct 26;16(21):3009. doi: 10.3390/polym16213009 (PMC11548547; doi:10.3390/polym16213009)
Supplement: Supplementary file 1 [file polymers-16-03009-s001.zip › polymers-3267887-supplementary.pdf]

Supplementary Material

On the Properties of Styrene–Maleic Acid Copolymer–Lipid Nanoparticles: A Solution NMR Perspective

Vladislav V. Motov [1,2], Erik F. Kot [1,3], Svetlana O. Kislova [4], Eduard V. Bocharov [1], Alexander S. Arseniev [1], Ivan A. Boldyrev [4], Sergey A. Goncharuk [1,*] and Konstantin S. Mineev [1,†,*]

[1] Shemyakin-Ovchinnikov Institute of Bioorganic Chemistry RAS, 117997 Moscow, Russia; motov.vladislave@gmail.com (V.V.M); erick.kot@gmail.com (E.F.K.); bon@nmr.ru (E.V.B.); aars@nmr.ru (A.S.A.)

[2] Moscow Center for Advanced Studies, Kulakova str. 20, Moscow 140829, Russia

[3] Faculty of Biology, Shenzhen MSU-BIT University, Shenzhen, 518172, China

[4] Frumkin Institute of Physical Chemistry and Electrochemistry, Russian Academy of Sciences, 119071, Moscow, Leninsky prospect 31, Russia; s.o.kislova@phyche.ac.ru (S.O.K.); i_boldyrev@mail.ru (I.A.B.)

[*] Correspondence: ms.goncharuk@gmail.com (S.A.G.), mineev@nmr.uni-frankfurt.de (K.S.M.)

[†] Current address: Institute of Organic Chemistry and Chemical Biology, Goethe University Frankfurt, 60438 Frankfurt am Main, Germany.

**DMPC/SMA-EA**		**DMPC/SMA-tau**	
**303 K**		**303 K**	
**r, nm**	2.78 ± 0.05	**r, nm**	2.90 ± 0.02
**λ**	5.17 ± 0.32	**λ**	9.37 ± 0.34
**313 K**		**313 K**	
**r, nm**	2.53 ± 0.04	**r, nm**	2.65 ± 0.02
**λ**	2.86 ± 0.13	**λ**	6.07 ± 0.19

Table S1. Parameters of approximation DMPC/SMA R(q) dependencies using ideal bicelle model with cylindrical rim.

DMPC/SMA-EA		DMPC/SMA-tau	
**q**	**phase transition temperature, K**	**q**	**phase transition temperature, K**
0.3	phase transition is not observed	0.2, 0.6	phase transition is not observed
0.6	not determined		
1.0	294.8 ± 0.4	1.0, 1.2, 1.4	not determined
1.5	296.5 ± 1.5	1.6	296.6 ± 0.9
1.8	296.3 ± 1.2	2.0	296.7 ± 0.8
2.0	295.5 ± 0.4	2.5	296.1 ± 1.1
**POPC/SMA-EA**		**DPPC/SMA-EA**	
1.5	not determined	1.5	311.3 ± 0.6
**DMPC/DMPG/SMA-EA q=2.0**		**DMPC/DMPG/SMA-tau q=2.0**	
**DMPC/DMPG ratio**	**phase transition temperature**	**DMPC/DMPG ratio**	**phase transition temperature**
80/20	DMPC: 295.4 ± 0.5<br>DMPG: 292.8 ± 1.1	80/20	DMPC: 295.5 ± 2.2<br>DMPG: 295.6 ± 1.1
70/30	DMPC: 295.9 ± 0.5<br>DMPG: 293.2 ± 1.0	70/30	DMPC: 294.5 ± 1.4<br>DMPG: 295.2 ± 1.5
60/40	DMPC: 296.6 ± 0.2<br>DMPG: 295.5 ± 0.6	60/40	DMPC: 291.3 ± 3.1<br>DMPG: 292.4 ± 1.9

Table S2. Phase transition temperatures in studied lipid/SMA samples. q value represents lipid/SMA mass ratio.

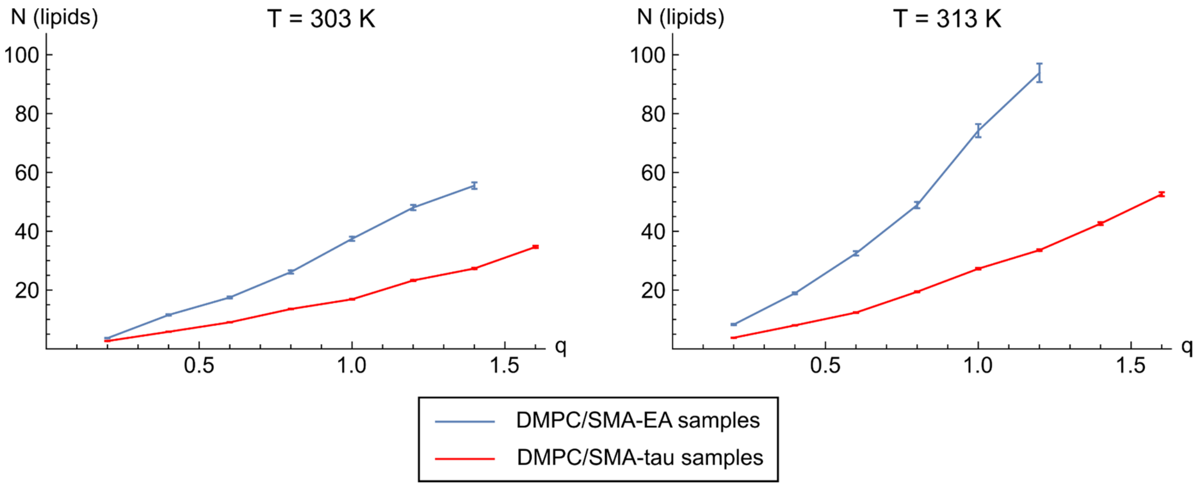

Figure S1. Number of lipid molecules at different q in observed DMPC/SMA-EA and DMPC/SMA-tau particles estimated with ideal bicelle model.

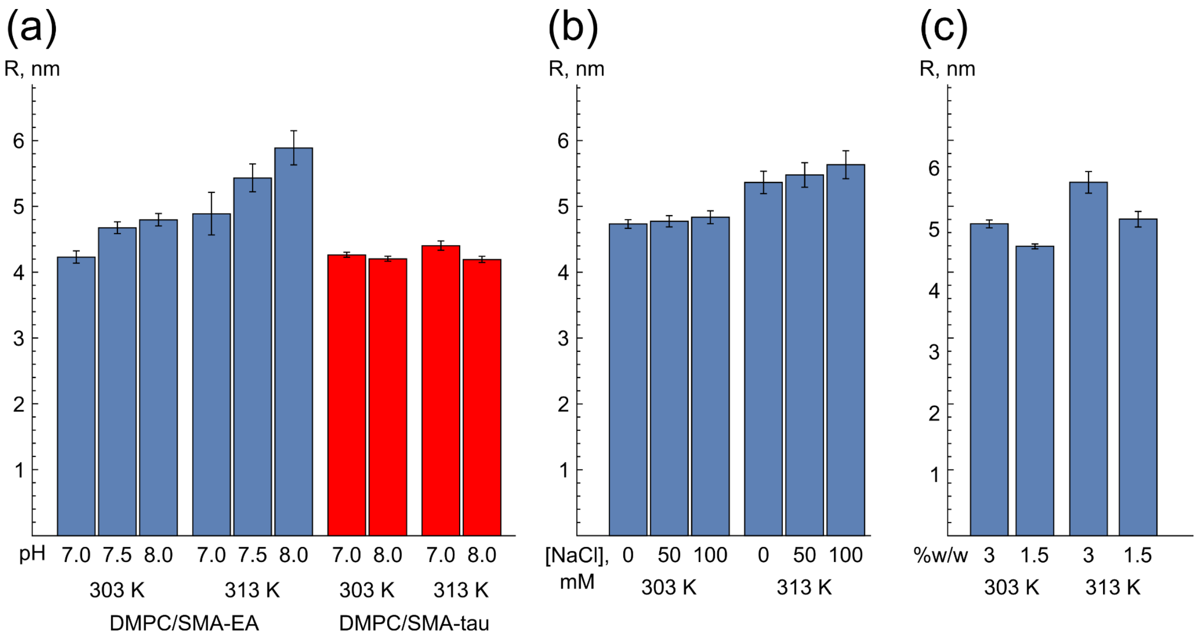

Figure S2. (**a**) Radii dependence on pH for DMPC/SMA-EA at q = 1.0 (w/v = 3%) and DMPC/SMA-tau at q = 1.0 (w/v = 3%) samples at 303 K and 313 K, (**b**) radii dependence on NaCl concentration for DMPC/SMA-EA q = 1.0, pH = 7.0 samples at 303 K and 313 K, (**c**) radii values for DMPC/SMA-EA q = 1.0, pH = 7.0, w/w 3% and 1.5% samples at 303 K and 313 K.

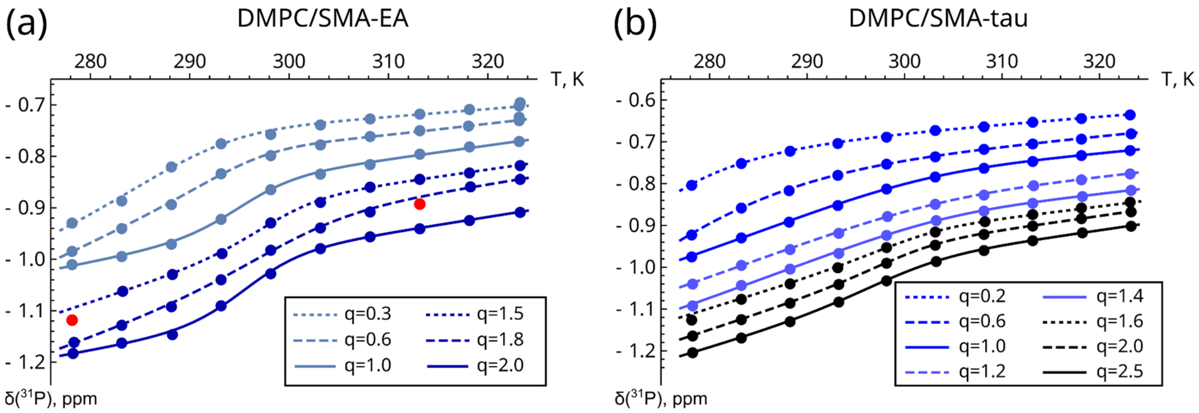

Figure S3. Temperature dependence of chemical shifts in ^{31}P spectra for DMPC/SMA-EA (**a**) and DMPC/SMA-tau samples (**b**). For DMPC/SMA-EA samples with q 1.0, 1.5, 1.8, 2.0 dependencies plots are shifted down by 0.025, 0.05, 0.1, and 0.12 ppm, respectively; for DMPC/SMA-tau samples with q 1.2, 1.4, 1.6, 2.0, 2.5 dependencies plots are shifted down by 0.05, 0.075, 0.1, 0.1, and 0.125 ppm, respectively**.**

(a)

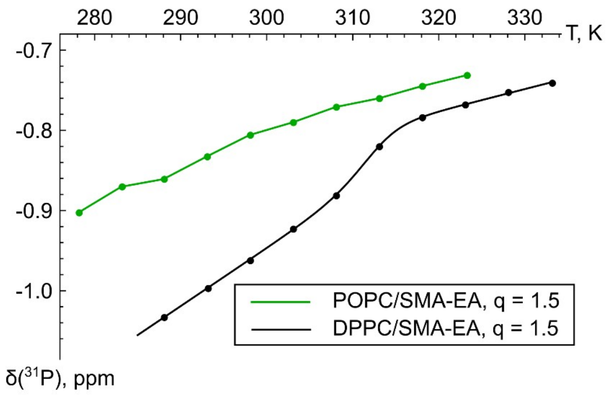

(b)

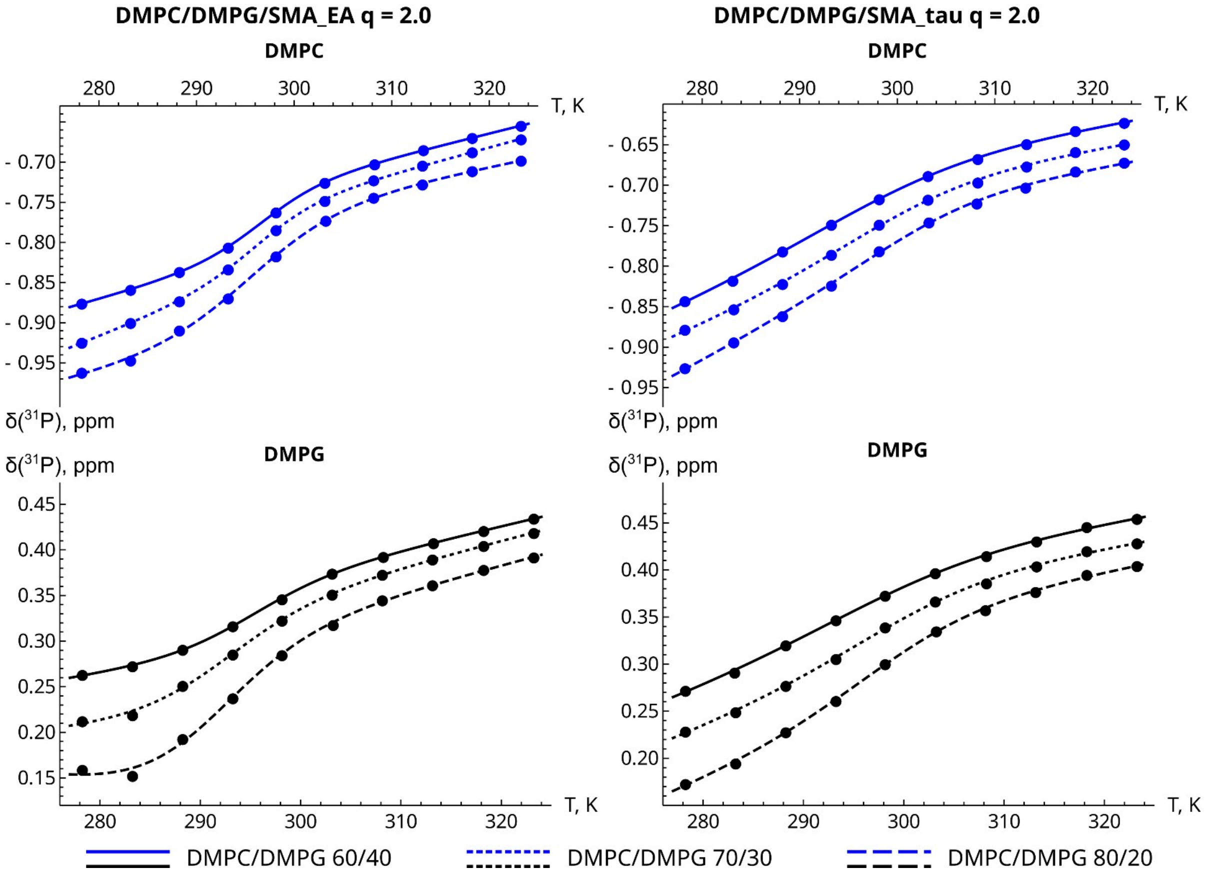

Figure S4. Temperature dependence of chemical shifts for (**a**) POPC/SMA-EA and DPPC/SMA-EA (q = 1.5) and (**b**) for DMPC/DMPG/SMA-EA and DMPC/DMPG/SMA-tau samples (q = 2) at DMPC/DMPG ratios 60/40, 70/30, 80/20.

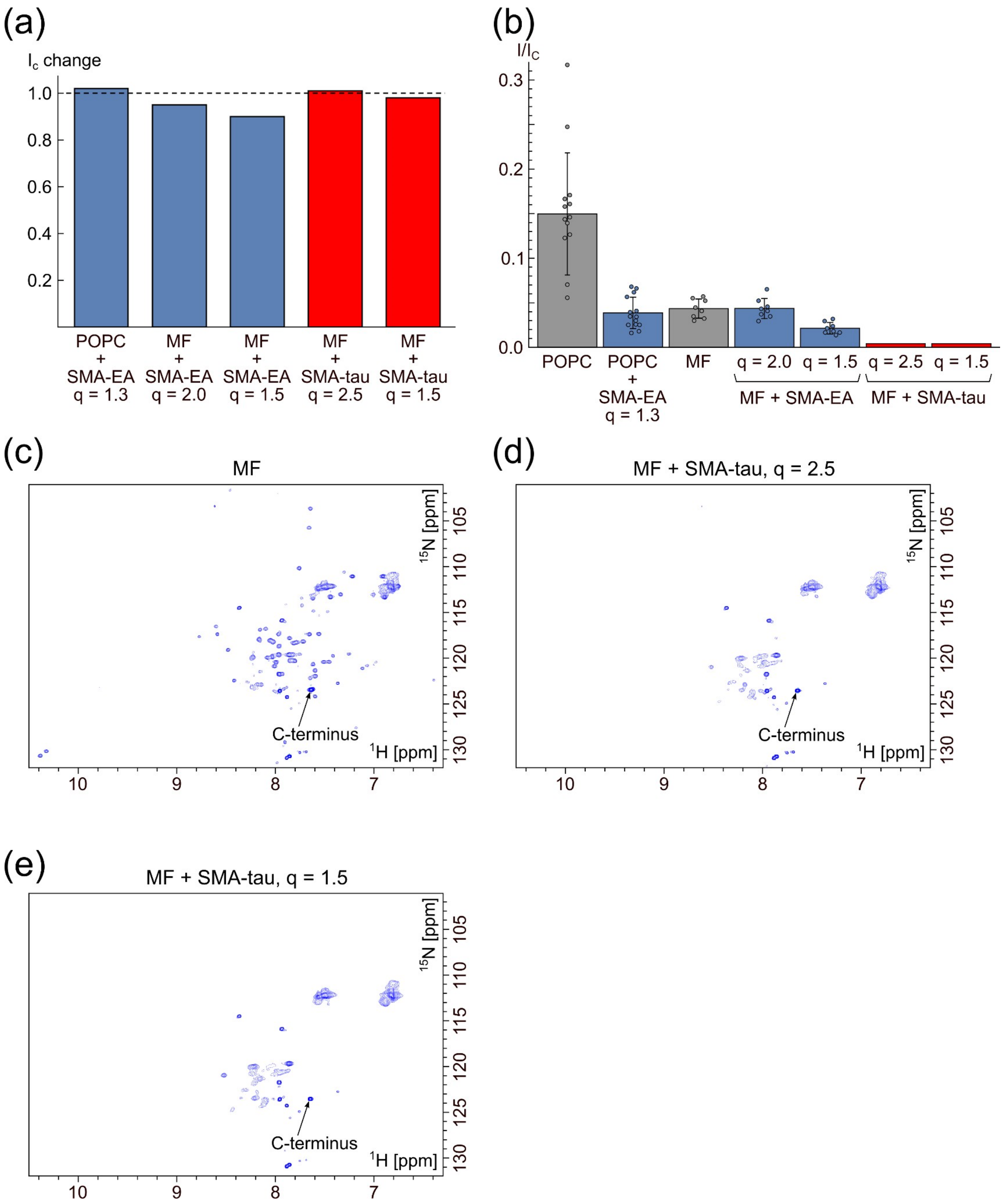

Figure S5. ^{1}H-^{15}N-HSQC spectra of p75-TM-ICD in different membrane mimetics. (**a)** Relative change in integral intensities of C-end amide groups signals after SMA addition; (**b)** Intensities of cross-peaks normalized to intensities of the C-terminal amide group signal; (**c**) E. coli membrane fraction (MF); (**d**), (**e**) - the mix of MF and SMA-tau at q = 2.5 and 1.5, respectively.
